# Isothermal and Non-Isothermal Crystallization Studies of Long Chain Branched Polypropylene Containing Poly(ethylene-*co*-octene) under Quiescent and Shear Conditions

**DOI:** 10.3390/polym9060236

**Published:** 2017-06-20

**Authors:** Zinan Zhang, Fengyuan Yu, Hongbin Zhang

**Affiliations:** Shanghai Key Lab of Polymer Dielectrics, Department of Polymer Science and Engineering, Advanced Rheology Institute, School of Chemistry and Chemical Technology, Shanghai Jiao Tong University, Shanghai 200240, China; zh1985zn@sjtu.edu.cn (Z.Z.); abelfengyuan@163.com (F.Y.)

**Keywords:** crystallization, polyolefins, elastomers, long chain branching, rheology, interfacial tension

## Abstract

Isothermal and non-isothermal crystallization behaviours of the blends of long chain branched polypropylene (LCB PP) and poly(ethylene-*co*-octene) (PEOc) with different weight ratios were studied under quiescent and shear flow using polarized optical microscopy (POM), differential scanning calorimetry (DSC), and rheological measurements. Experimental results showed that the crystallization of the LCB PP/PEOc blends were significantly accelerated due to the existence of the long chain branches (LCBs), the blends being able to rapidly crystallize even at 146 °C. The addition of PEOc that acts as a nucleating agent, could also increase the crystallization rate of LCB PP. However, the crystallization rate of LCB PP was reduced when the PEOc concentration was more than 60 wt %, showing a retarded crystallization growth mechanism. The morphology of the binary blend was changed from a sea-island structure to a co-continuous phase structure when the PEOc concentration was increased from 40 to 60 wt %. In comparison with linear isotactic iPP/PEOc, the interfacial tension between LCB PP and PEOc was increased. In addition, flow-induced crystallization of LCB PP/PEOc blends was observed. Possible crystallization mechanisms for both LCB PP/PEOc and iPP/PEOc blends were proposed.

## 1. Introduction

Isotactic polypropylene (iPP) is one of the leading and fast growing thermoplastic resins widely used in automotive, construction, and industrial applications. The outstanding chemical and physical properties, such as excellent chemical resistance, easy processability, low density, high tensile modulus, and low cost, make iPP a commercially important semicrystalline polymer. Commercially, iPP is synthesized by well-established techniques, including Ziegler-Natta and metallocene catalysts. However, in general, the prepared iPP exhibits relatively low melt strength and poor low-temperature impact resistance because of the presence of highly linear chains and relatively narrow molecular weight (*M*_w_) distributions. This limits its applicability in applications such as blow molding, thermoforming, extrusion coating, foaming, and certain other engineering fields which require high impact performance.

So far, several strategies have been proposed to improve the melt strength of iPP. For example, increasing the *M*_w_, broadening the *M*_w_ distribution [1], or introducing long chain branches (LCBs) [2,3], are some of the methods adopted to improve the melt strength. Among them, the most efficient way is to introduce LCBs onto the backbone of iPP [4]. Until now, some approaches have been developed to synthesize long chain branched polypropylene (LCB PP), such as reactive extrusion [4,5,6,7,8,9,10,11,12] and electron beam irradiation [13,14]. Furthermore, one of the extensively used methods to improve the toughness of iPP is by blending it with an elastomer, including ethylene propylene rubber (EPR) [15,16,17], ethylene propylene diene terpolymer (EPDM) [18,19,20,21,22] and poly (styrene-ethylene/butylene-styrene) (SEBS) triblock copolymer [23,24,25,26,27]. More recently, poly (ethylene-*co*-octene) (PEOc) has been used as an impact modifier for iPP [28,29,30,31]. Compared with traditional elastomers, PEOc not only has superior toughness, high shear sensitivity and high melt strength, but also exhibits good compatibility with iPP. The narrow *M*_w_ distribution and homogeneous octane distribution in PEOc prevent warpage in injection molding and extrusion. Moreover, its good flowability can improve the dispersion effect [32,33,34]. Following the addition of PEOc, several researchers have reported the significant improvement of the impact strength of iPP [34,35,36].

The crystallization behaviour of semicrystalline polymer materials plays an important role in determining the mechanical properties of the resulting plastic products. Apart from affecting the rheological properties, it is recognized that the presence of LCBs may also influence the crystallization properties. In our previous study, reported elsewhere [8], we had reported that the introduction of LCBs onto the backbone of iPP significantly affected its flow-induced crystallization behaviour. The LCB PP resin with a high level of branched chains showed accelerated crystallization rate compared to that with a low level of branched chains. In addition, the crystallization of LCB PP was more sensitive to shear flow than that of linear PP during the induction period even at low shear rates. Similar behaviour has been reported for other crystalline polymers with LCB structures, such as LCB polylactide and LCB polycarbonates [37,38,39].

Several researchers have analyzed in detail the crystallization behaviour of iPP/PEOc blends [40,41,42,43,44]. In our previous study, we demonstrated that the presence of the highly viscous elastomer, PEOc, tended to promote the nucleation of the supercooled iPP melting at the interface of two-phases due to their long relaxation behaviour in shear flow field [45,46]. Moreover, polymers usually undergo complicated flow fields during processing. Therefore, the understanding of the crystallization process of polymers under the effect of flow makes it possible to control and predict the final morphologies and properties of the polymers [47,48,49,50]. Earlier, we investigated the effect of shear flow at various shear rates, shear time, and shear strains on the crystallization of polyolefin melts [8,51,52,53]. However, although the crystallization of PP/PEOc blends has been studied [28,29,30,31,32,33,34,35,36], we know of no reports on the crystallization in LCB PP/PEOc blends under either quiescent or shear states. 

Herein, we describe our investigations of the isothermal and non-isothermal crystallization behaviour of two LCB PP/PEOc blends under quiescent and shear flow, and compare the crystallization behaviours of these blends under these two conditions. The combined influences of LCBs and PEOc on the crystallization are evaluated, based on the crystallization kinetics. In addition, the changes in morphology of the PP/PEOc binary immiscible systems are also discussed. The interfacial tensions for the LCB PP/PEOc and the iPP/PEOc blends are estimated by using the Palierne Model [54]. Based on the systematic studies, a possible mechanism for the crystallization of LCB PP/PEOc blend is proposed.

## 2. Materials and Methods 

### 2.1. Materials 

The polymeric materials used were iPP (T300, Sinopec Shanghai Petrol Chemical Co., Ltd., Shanghai, China), with a melt flow rate of 3.0 g/10 min (230 °C, 2.16 kg) and a density of 0.91 g cm^−3^, and PEOc (Engage 8150, Dow Chemical Co., Ltd., Midland, MI, USA) a random copolymer with 25% octene, a melt flow rate of 0.44 g/10 min (190 °C, 2.16 kg), 55 °C melting peak temperature (cooling rate 10 °C/min) and a density of 0.87 g cm^−3^. Multifunctional monomers, namely 2,5-dimethyl-2,5-di(*tert*-butylperoxy)hexane peroxide and pentaerythritol triacrylate (PETA), were purchased from Sinopharm Chemical Reagent Co., Ltd., Shanghai, China. In addition, to stabilize iPP samples during preparation of LCB PP, an antioxidant (Inganox 1010, Ciba Specialty Chemicals Co., Ltd., Basel, Switzerland) was used.

### 2.2. Preparation of LCB PP

LCB PP was prepared by the modification of the commercially purchased iPP by way of reactive extrusion processing using a twin screw extruder (ZE 25A, Berstorff GmbH, Hanover, Germany) equipped with a screw of diameter 25 mm and a length/diameter ratio (L/D) of 41. We have reported details on the reactive extrusion processing used here previously [7,8]. Before the reactive extrusion process, 1.5 phr PETA was dissolved in acetone at a concentration of 20 wt %. Following that, the prepared PETA solution was mixed with 100 phr PP, 0.2 phr Irganox 1010, and 0.1 phr peroxide. The mixture was added into the extruder via the feed port, with an extrusion temperature of 180 °C and a twin screw rotational speed of 150 rpm. The resulting unpurified sample was dissolved in xylene at 120 °C for 10 min, and then precipitated in acetone at room temperature. Such repeated procedure was made to completely remove any unreacted PETA monomer and co-polymerized PETA [7]. Finally, the purified LCB PP was dried in a vented oven at 50 °C overnight. The weight fraction of LCBs in the obtained LCB PP sample was 37% [8].

### 2.3. Preparation of LCB PP/PEOc Blends

Mixtures of LCB PP and PEOc, containing 20, 40, 60 and 80 wt % of PEOc, were prepared by melt-compounding using a HAAKE internal mixer of chamber volume 60 cm^3^ (Rheocord 90, HAAKE GmbH, Vreden, Germany). During the mixing process, the temperature was kept at 180 °C, and the mixer was rotated at a constant rotor speed of 75 rpm. The resulting blends were compression-molded into sheets using a hot-press (XLB-D, Rubber Machinery Factory, Guilin, China), under a pressure of 10 MPa at 190 °C. For reference, blends of iPP and PEOc, containing 20, 40 and 60 wt % of PEOc, were also prepared in the same way.

### 2.4. Polarized Optical Microscopy (POM)

The quiescent isothermal crystallization behaviours of the LCB PP/PEOc and the iPP/PEOc blends were observed using a Leica DMLP (Leica Microsystems GmbH, Wetzlar, Germany) POM, equipped with an automatic hot-stage (Linkam TH960, Linkam Scientific Instruments Ltd., Surrey, UK) with a precision of 0.1 °C. A thin film of thickness 0.1 mm was prepared for each observation. During the POM observation, the blends were heated to a temperature of 180 °C and held at that temperature for 5 min to completely erase any thermal history. Subsequently, the blends were cooled down to the isothermal crystallization temperature (140 °C) at a cooling rate of 50 °C min^−1^.

### 2.5. Differential Scanning Calorimetry (DSC)

Furthermore, to substantiate the results derived from POM, the quiescent isothermal crystallization behaviours of the LCB PP/PEOc and the iPP/PEOc blends were further investigated by using DSC (Perkin Elmer PYRIS-1, San Diego, CA, USA). For this, 5 mg of the samples were quickly heated up at 50 °C min^−1^ until the temperature reached 180 °C. The blends were held at that temperature for 5 min to completely erase the thermal history. After that, the samples were cooled down to the isothermal crystallization temperature (140 °C) at 50 °C min^−1^.

In addition to the analysis of quiescent isothermal crystallization behaviour, the quiescent non-isothermal crystallization behaviours of the blends were also analyzed. 5 mg of the samples were held at 180 °C for 5 min, followed by cooling down to ambient temperature at various cooling rates of 1, 3 and 5 °C min^−1^. All the experimental processes were performed under a nitrogen atmosphere.

### 2.6. Rheological Measurements

Rheological experiments were carried out on a Bohlin Gemini 200HR rheometer (UK) with parallel plate geometry (25 mm in diameter and 1 mm in gap). The dynamic mechanical properties of the LCB PP/PEOc and the iPP/PEOc blends were investigated by performing isothermal dynamic frequency sweeps (0.01–100 rad s^−1^) at 180 °C, at a given strain (γ = 1%) in the linear viscoelastic regime. Before testing, the blends were held at 180 °C for 5 min to erase the thermal history.

To investigate the isothermal crystallization process, isothermal dynamic rheological experiments were performed on the melts. Prior to measurements, all the samples were heated up to 180 °C and held at this temperature for 5 min. Subsequently, the samples were cooled down to the isothermal crystallization temperatures (140 and 146 °C) at a cooling rate of 50 °C min^−1^. Following that, a constant dynamic frequency of 1 rad s^−1^ was applied in the linear viscoelastic regime at this temperature.

Furthermore, to investigate the flow-induced non-isothermal crystallization process, the samples were initially heated to 180 °C and held at this temperature for 5 min. Subsequently, while cooling down the samples to ambient temperature at various cooling rates of 1, 3 and 5 °C min^−1^, the samples were operated under a constant uniform shear flow at a rate of 0.1 s^−1^.

### 2.7. Scanning Electron Microscopy (SEM)

After blending at 180 °C, the LCB PP/PEOc and the iPP/PEOc blends were quickly quenched in liquid nitrogen for 5 min to freeze the morphology, and then fractured into two halves immediately. Contrast between the continuous matrix and the dispersed phase was obtained by selective extraction of the PEOc component. The fractured surfaces were etched with xylene at room temperature overnight, followed by vacuum drying to remove the residual solvent. Finally, samples were sputter coated with a thin layer of gold and observed using a scanning electron microscope (SEM) (JSM-7401F, JEOL Ltd., Tokyo, Japan).

It should be noted that, since PEOc is the matrix phase for the samples of PP/PEOc-20/80 and 40/60, the structures of these samples would be destroyed and became loose after etching. thus, here the samples of PP/PEOc-80/20 and 60/40 were chosen for comparison in the SEM observation. 

## 3. Results and Discussion

In our opinion, the blends with the weight ratio of PP/PEOc-80/20 and 60/40 may have quite similar crystallization behaviours. In these two blends, PP is the matrix phase and PEOc is the separated phase, and the difference in content of component cannot lead to an essential change. However, when the PEOc goes to 60 wt %, the phase reversion happens, and PEOc becomes the matrix phase. The phase behaviour is closely related to the crystallization behaviour. The phase reversion may cause some unusual crystallization behaviours. In addition, for the PP/PEOc-20/80 blends, the content of crystalline polymer is very low. At this weight ratio, the POM experiments are also difficult to carry out. Based on above consideration, and in order to make the discussion convenient, we thus chose the samples of PP/PEOc-80/20 and 40/60 to compare and discuss in the following for most methods.

### 3.1. POM Observations

**Figure 1:**
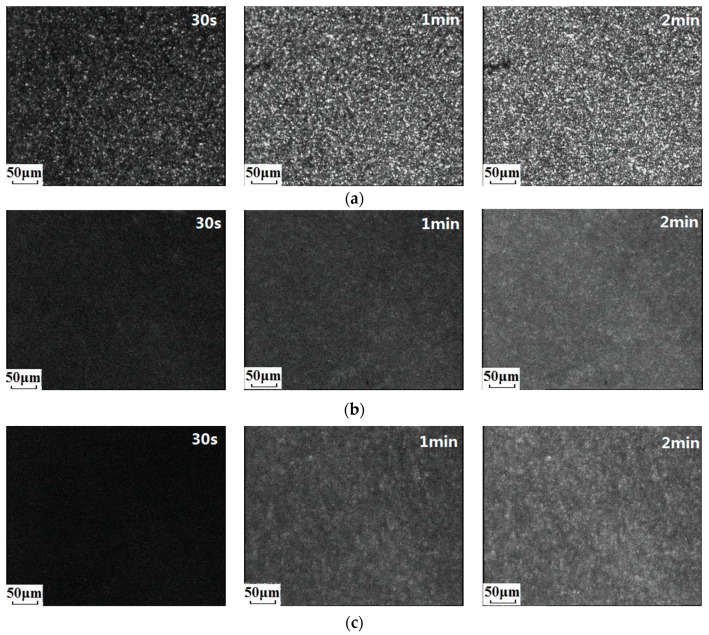
Polarized optical micrographs of (**a**) LCB PP; (**b**) LCB PP/PEOc-80/20; and (**c**) LCB PP/PEOc-40/60 during isothermal crystallization at 140 °C for 30 s, 1 min and 2 min, respectively, left to right.

The POM of the LCB PP/PEOc and the iPP/PEOc blends were analyzed to investigate their isothermal crystallization behaviours. Figure 1a shows the polarized optical micrographs of the LCB PP sample, during isothermal crystallization at 140 °C for various time durations, namely, 30 s, 1 min and 2 min. Tiny crystal nuclei were initiated in large numbers in the field of view, and no perfect spherulites could be found, with a high crystal nuclei density being observed (Figure 1a). Similar results were observed for the LCB PP/PEOc blends in Figure 1b,c, except the crystallization was delayed. However, the nucleation in all three samples could be observed in less than 1 min during the isothermal process, although the growth of spherulites was not distinct in 1 min at the magnification and sample thickness (overlapping spherulites) used. Tian et al. [7] proved that the reactive extrusion process could make the LCBs graft onto the backbone of iPP without causing its degradation. The unreacted PETA monomer and co-polymerized PETA were soluble and were removed by solvents during the purification process we used. The LCBs in the blends should thus be responsible for the crystallization behaviours of LCB PP as observed by POM. The introduction of LCBs increased the number of entanglement points which could help PP to form stable crystallization precursors of primary nuclei for crystallization of extended chain crystals, consequently increasing the crystal nuclei density and the crystallization kinetics of PP [48]. Promotion of PP to form stable crystallization precursors of primary nuclei for crystallization will be further verified by the analysis below based on isothermal DSC measurements.

**Figure 2:**
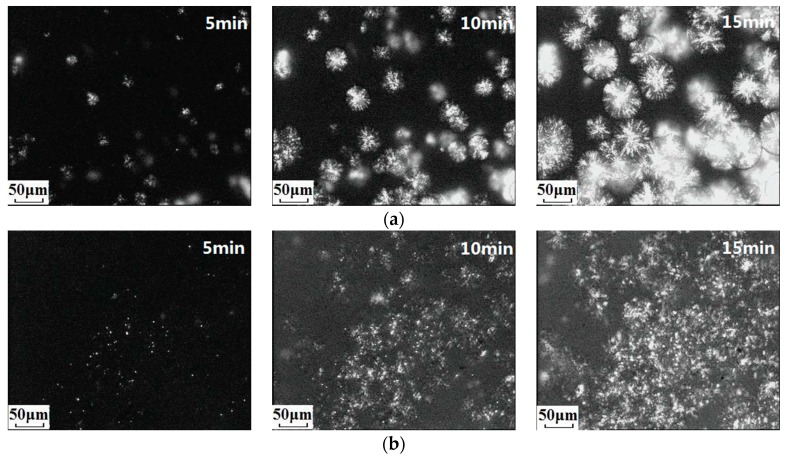
Polarized optical micrographs of (**a**) iPP/PEOc-80/20; and (**b**) iPP/PEOc-40/60 during isothermal crystallization at 140 °C for 5, 10 and 15 min, respectively, left to right.

The iPP/PEOc blends had a much lower crystal nuclei density and slower crystallization rate than LCB PP/PEOc blends, as shown in Figure 2, recorded at the same temperatures determined but at 5, 10 and 15 min, respectively. The number of the crystal nuclei can be readily quantitatively calculated in the same field of view areas. There were only about 54 crystal nuclei for the iPP/PEOc-80/20, while about 70 crystal nuclei could be observed for the iPP/PEOc-40/60. The spherulites could be found in 5 min, and the obvious growth of spherulites could be observed as time goes on. With the increase of the PEOc content, the spherulites became imperfect, and the crystal nuclei density increased slightly. 

### 3.2. Isothermal Dynamic Rheological Experiments

**Figure 3:**
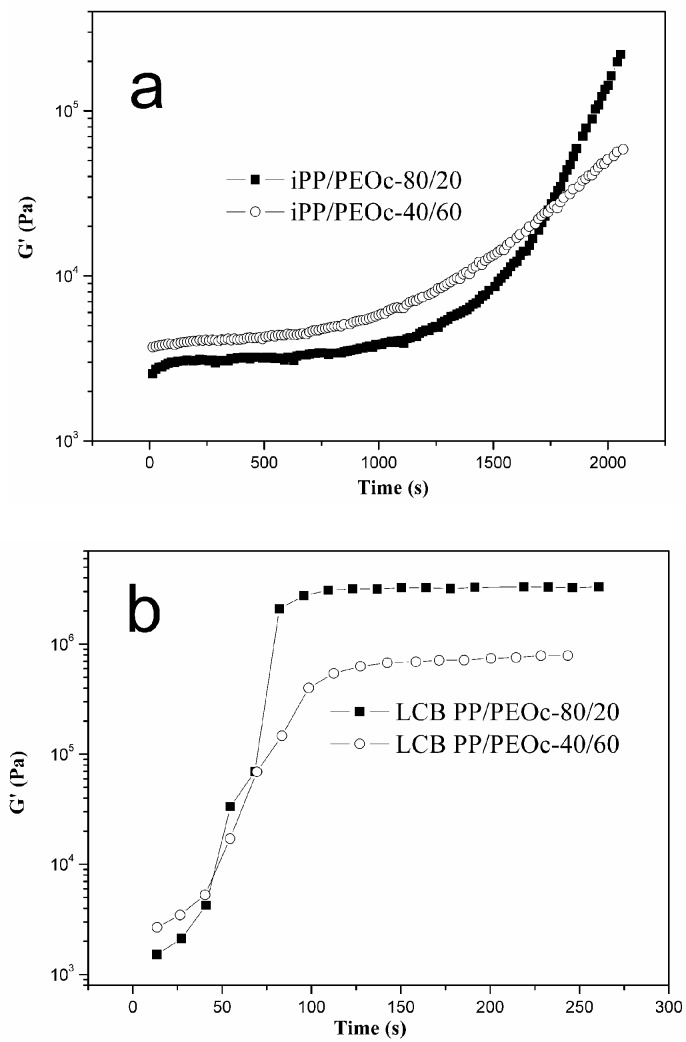
Storage modulus development curves at 140 °C for (**a**) iPP/PEOc-80/20 and iPP/PEOc-40/60; and (**b**) LCB PP/PEOc-80/20 and LCB PP/PEOc-40/60 blends.

Isothermal dynamic rheological experiments were also performed to analyze the crystallization behaviours of the iPP/PEOc and the LCB PP/PEOc blends. Figure 3 shows the time dependence of the storage modulus, G’, for iPP/PEOc and LCB PP/PEOc 80/20 and 40/60 blends at 140 °C. Sharp increases in G’ for all the LCP PP/PEOc blends were observed when the crystallization of PP occurred. For the iPP/PEOc blends (Figure 3a), the G’ value of iPP/PEOc-40/60 started to increase earlier than that of iPP/PEOc-80/20, which indicated that the blends with a higher PEOc content had a shorter onset time of crystallization. This could be attributed to the role of PEOc, which acted as a nucleation agent, thus promoting the crystallization of blends. We have previously demonstrated this in our earlier study [45]. For the LCB PP/PEOc blends (Figure 3b), the onset time of crystallization became much shorter than that of iPP/PEOc, which was due to the synergistic acceleration of the LCBs on the nucleation rate of PP. Our previous work [8] showed that the grafted PETA on the branched chain acted as a heterogeneous nucleating agent for PP crystallization, by increasing the number of entanglements restricting the segmental motion during melting. As a result, the crystallization capability of LCB PP can be significantly enhanced rather than weakened, as the branched chain level is increased. This enhancement effect was confirmed, as evidenced from the increase in the plateau modulus value with the LCB PP content, shown in Figure 3b. However, the enhancing effect of the PEOc component on the crystallization nucleation seemed to be much weaker than that of LCBs, i.e., the increase in PEOc concentration seemed to have almost no influence on the onset time of crystallization of the LCB PP/PEOc blends. Moreover, a plateau modulus was observed in Figure 3b, which should be attributed to the slippage between the parallel plate geometry of the instrument and the LCB PP/PEOc blend with a high crystallinity. Thus, the results of the isothermal dynamic rheological experiments in this study were in good agreement with the POM observations.

**Figure 4:**
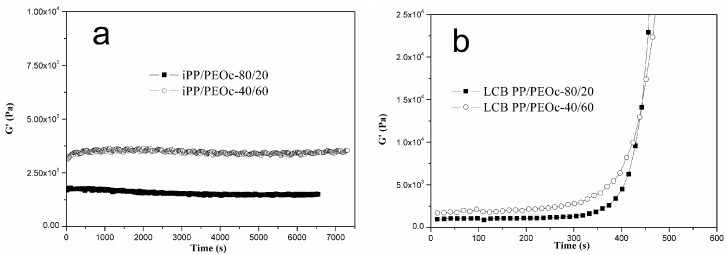
Storage modulus development curves at 146 °C for (**a**) iPP/PEOc-80/20 and iPP/PEOc-40/60; and (**b**) LCB PP/PEOc-80/20 and LCB PP/PEOc-40/60 blends.

When the isothermal crystallization temperature was increased to a higher value, the effect of LCBs on the crystallization of PP was more evident. Figure 4 shows the time evolution of G’ for the iPP/PEOc and the LCB PP/PEOc blends at 146 °C. In the case of iPP/PEOc blends (Figure 4a), no crystallization behaviour could be observed in either sample, even after holding it at 146 °C for more than 6000 s. On the contrary, the LCB PP/PEOc blends crystallized with a sharp increase in G’ at about 400 s (Figure 4b). This fact further substantiated the strong enhancing effect of LCBs on the crystallization of PP.

### 3.3. Isothermal DSC Measurements

The crystallization kinetics of the blends were analyzed by using a classical Avrami equation [55,56,57], as given in Equation (1):

(1) $$1-X_{c}=\exp(-kt^{n})$$

where *X*_c_ is the relative crystallinity, *n* the Avrami exponent, *k* the kinetic constant, and *t* the crystallization time. The parameters, *n* and *k*, can be used to qualitatively interpret the nucleation mechanism, morphology, and the overall crystallization rate of the polymer. The value of the Avrami exponent, *n*, depends on the nucleation mechanism and growth dimension. The kinetic constant, *k*, is a function of the nucleation and the growth rate. The relative crystallinity is the ratio of the true crystallinity, *X*_c_ (*t*), to the maximum crystallinity, *X*_e_ = *X*_c_ (*t* → ∞):

(2) $$X_{c}=\frac{X_{c}(t)}{X_{e}};\;0<X_{c}\leq 1$$

In order to deal conveniently with the operation, Equation (1) is usually rewritten in the double logarithmic form as follows:

ln[−ln(1 − *X_c_*)] = ln(*k*) + *n* ln(*t*) (3)

Here, the values of *n* and *k* could be directly obtained using Equation (3), by calculating the slope and intercept of the best-fit line, respectively.

**Figure 5:**
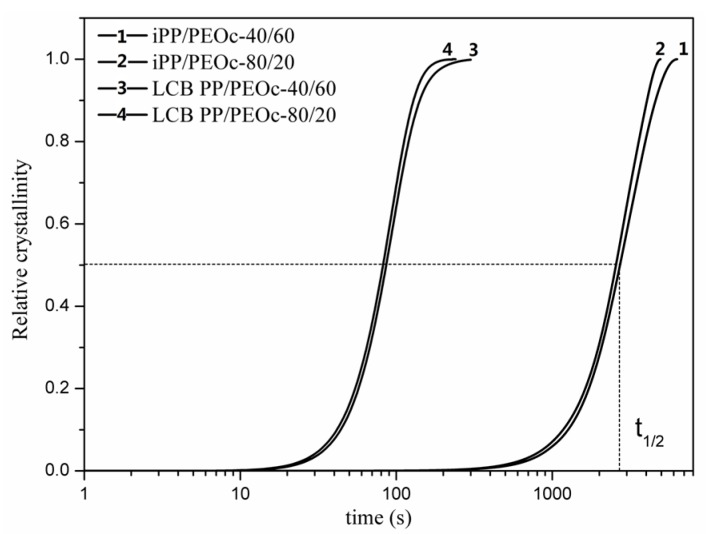
Relative crystallinity of iPP/PEOc and LCB PP/PEOc blends undergoing isothermal crystallization at 140 °C.

**Figure 6:**
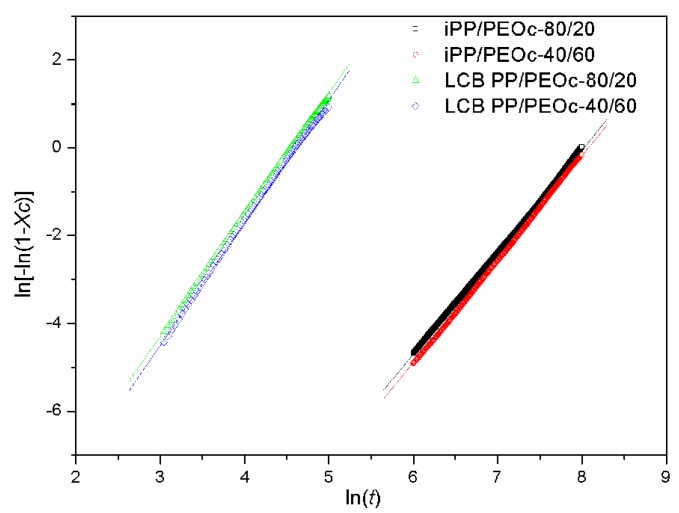
The Avrami plots for iPP/PEOc and LCB PP/PEOc blends isothermally crystallized at 140 °C.

Figure 5 shows the integrated curves of the relative degree of crystallinity in the course of isothermal crystallization at 140 °C. The corresponding crystallization half times, *t*_1/2_, were estimated directly from Figure 5. The Avrami plots for iPP/PEOc and LCB PP/PEOc blends isothermally crystallized at 140 °C are shown in Figure 6. Obviously, the Avrami curves for the iPP/PEOc and the LCB PP/PEOc blends were quite different. Table 1 summarizes the Avrami exponent *n*, and kinetic constants *k*, alone with *t*_1/2_ for the selected blend compositions and crystallization at 140 °C. The Avrami exponent *n* is the mechanism constant, whose value depends on the type of nucleation and growth dimension. The *n* = 3 indicates three-dimensional spherulitic growth from instantaneous nuclei (athermal nucleation). If *n* equals 2, it means that crystallization occurred in two-dimensional aggregates (like axialites or lamellar aggregates) with an instantaneous nucleation [58]. The exponent *n* for the iPP/PEOc blends was ca. 2.3, which is close to 2, while the exponent *n* for the LCB PP/PEOc blends was ca. 2.8, close to 3, a value that corresponds to the heterogenous nucleation of a three-dimensional growth. The fact that the LCB PP/PEOc blends exhibited much larger *k* values and much shorter *t*_1/2_ than iPP/PEOc is in good agreement with the rheological results (Figure 3 and Figure 4). These results signify that the crystallization rate of the samples were increased significantly in the presence of the LCBs. 

As for the influence of the PEOc on the crystallization is concerned, the PEOc acted as a nucleation agent, slightly promoting the crystallization process of the blends. On the other hand, the crystallization rate of both iPP/PEOc and LCB PP/PEOc blends was reduced with increase in the content of the PEOc. This suggests the possibility of a retarded growth mechanism due to the dispersion of a large amount of the PEOc in the matrix. The decrease in *n* and *k* values along with the extended *t*_1/2_ in Table 1 implies that the addition of excessive amounts of PEOc may restrain the LCB PP mobility, hindering the growing front of the PP spherulites and thus reducing the crystallization rate of PP.

### 3.4. Non-Isothermal DSC Measurements

**Figure 7:**
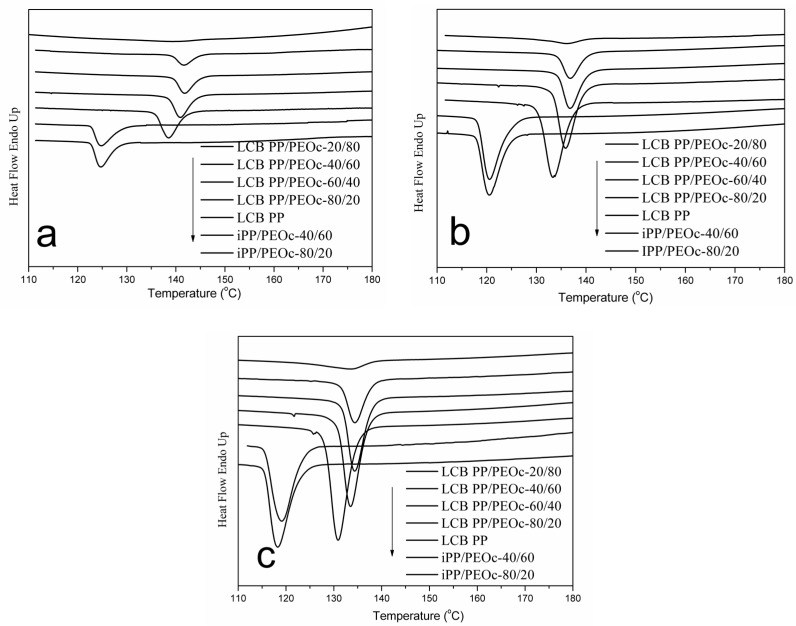
DSC curves for LCB PP/PEOc and iPP/PEOc blends at a cooling rate of (**a**) 1 °C min^−1^; (**b**) 3 °C min^−1^; and (**c**) 5 °C min^−1^.

**Figure 8:**
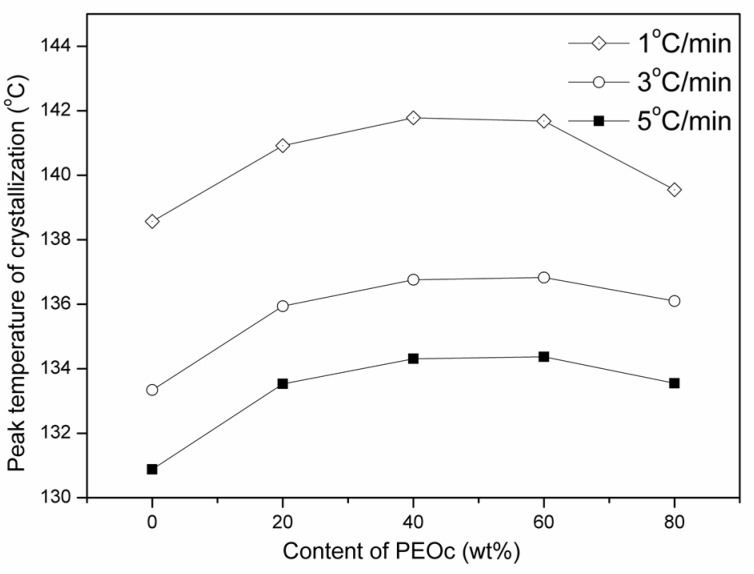
Peak temperatures of crystallization for the LCB PP/PEOc blends as a function of PEOc content at various cooling rates.

Figure 7 shows the non-isothermal crystallization behaviours of all of the LCB PP/PEOc and the iPP/PEOc blends, investigated by using DSC. It was found that, at a given cooling rate, the LCB PP/PEOc blends had a much higher peak temperature of crystallization than that of the iPP/PEOc blends, showing clear accelerated crystallization kinetics. Figure 8 shows the peak temperature of crystallization for the LCB PP/PEOc blends as a function of the PEOc content at the various cooling rates. The peak crystallization temperature was increased with the content of PEOc until it reached 60 wt %. We suggest that the addition of PEOc in LCB PP accelerate the nucleation process and increase the nucleation density due to the heterogeneous nucleation at the two-phase interface. However, the PEOc component can also inhibit the growth of spherulites, as revealed in the abovementioned isothermal crystallization processes. For the LCB PP/PEOc blends, this inhibition effect became significant, especially when the content of PEOc was larger than 60 wt %.

Additionally, Figure 8 shows the influence of cooling rate on the crystallization process. As the cooling rate was changed from 1 to 5 °C min^−1^, the crystallization peak and the corresponding onset temperature of crystallization shifted toward a lower temperature. This is due to that crystallization is a process of polymeric chains and segments rearranging and entering into a crystal lattice. There exists a lag period between the crystallization process and temperature fall. As a result, the non-isothermal crystallization tends to be slower than the drop of the temperature. The lag period increased with the cooling rate, becoming much more evident [29,42], as shown in Figure 7c and Figure 8, for a cooling rate of 5 °C min^−1^.

### 3.5. Non-Isothermal Steady Shear Rheological Experiments

**Figure 9:**
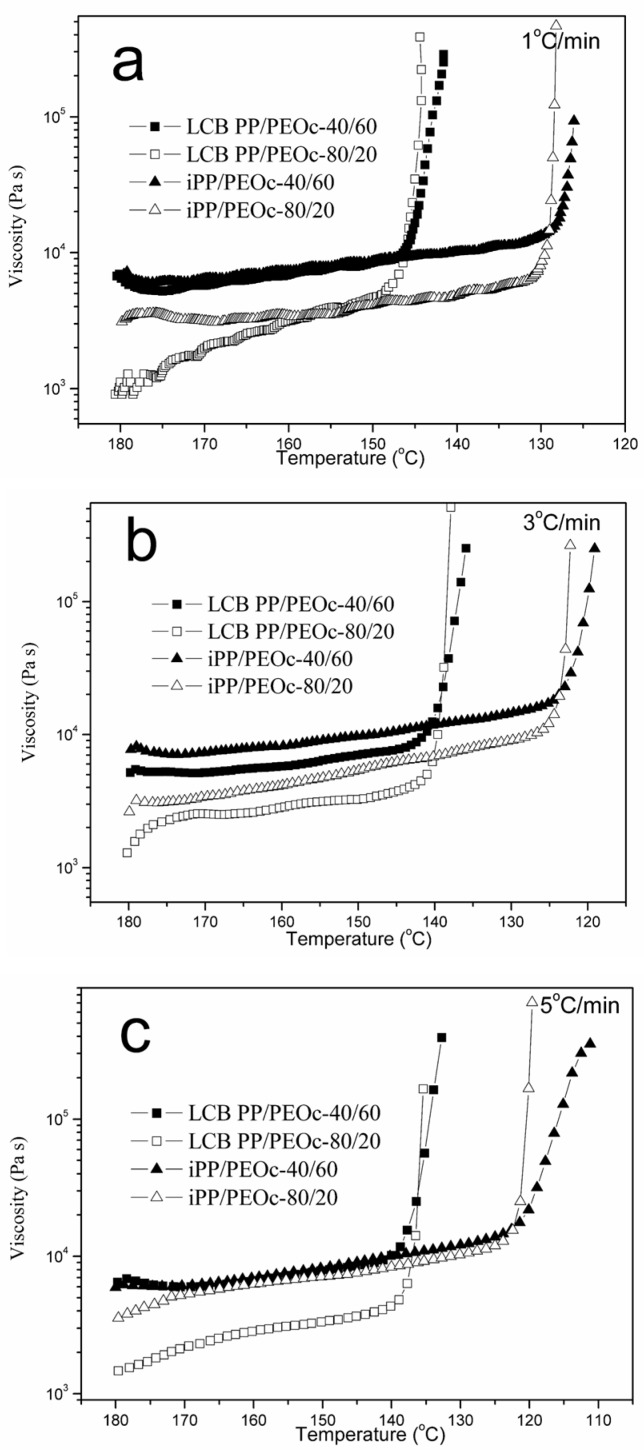
Viscosity development curves of LCB PP/PEOc and iPP/PEOc blends at the cooling rates of (**a**) 1 °C min^−1^; (**b**) 3 °C min^−1^; and (**c**) 5 °C min^−1^, at a shear rate = 0.1 s^−1^.

**Figure 10:**
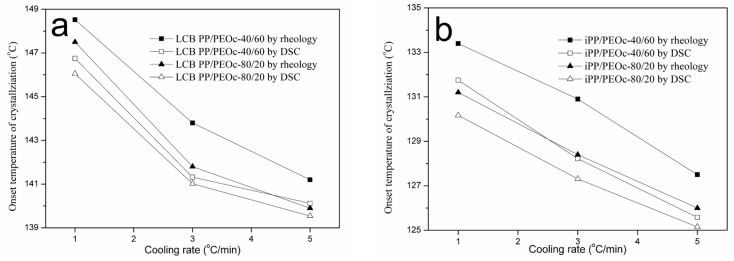
Plot showing the onset temperature of crystallization for (**a**) LCB PP/PEOc blends and (**b**) iPP/PEOc blends as a function of cooling rate, as determined from the rheological and DSC experiments.

Complementing the DSC measurements, non-isothermal cooling steady shear rheological experiments were also carried out, as shown in Figure 9. Here, the sharp increase in the viscosity corresponds to the crystallization of PP in the blends. As expected, the LCB PP/PEOc blends had much higher onset temperatures than those of the iPP/PEOc blends, and their onset temperatures of crystallization shifted toward lower temperatures as the cooling rate increased. Moreover, as shown in Figure 10, the crystallization onset temperatures of blends calculated from the rheological experiments were higher than those determined from the DSC measurement. This significant increase in the onset temperature could be attributed to the fact that the crystallization process of PP was promoted by the flow field imposed in the rheological experiments, which made it possible for PP to crystallize at a higher temperature. The observed experimental results of flow-induced crystallization were consistent with that demonstrated in our previous work [8,51,52,53].

### 3.6. Morphology Observations

**Figure 11:**
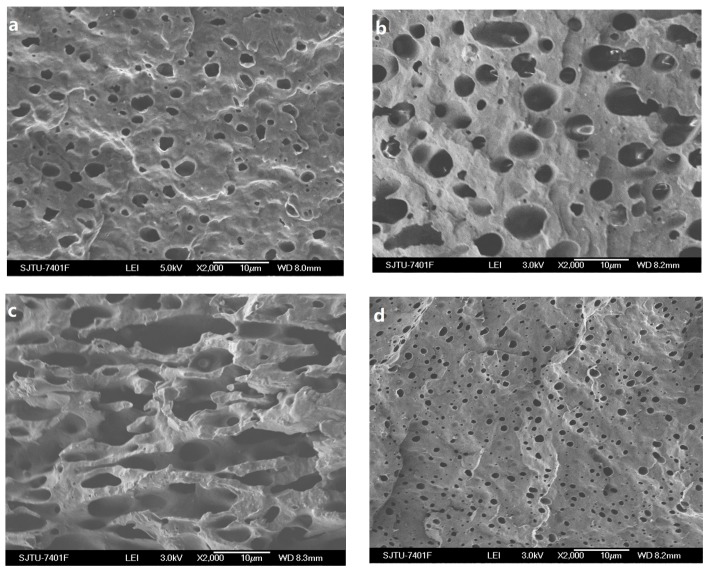

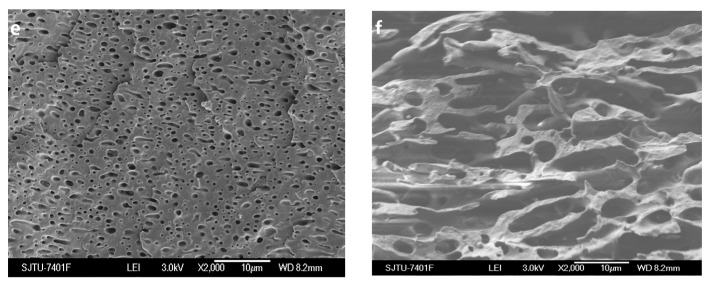
SEM images showing the fracture surfaces of (**a**) LCB PP/PEOc-80/20; (**b**) LCB PP/PEOc-60/40; (**c**) LCB PP/PEOc-40/60; (**d**) iPP/PEOc-80/20; (**e**) iPP/PEOc-60/40 and (**f**) iPP/PEOc-40/60 blends (etched by xylene at room temperature).

Figure 11 shows the SEM images of the LCB PP/PEOc and the iPP/PEOc blends. The fractured surfaces of the blends were etched by xylene to dissolve the PEOc component. When the PEOc content was less than 60 wt %, the blend exhibited a typical droplet-matrix structure with a continuous phase of PP. With increasing PEOc content, the average size of the dispersed phases increased. When the PEOc content reached 60 wt %, the morphology of blends changed from a droplet-matrix structure to a co-continuous phase structure. In addition, at the same weight ratio, the average sizes of the dispersed phase in the LCB PP/PEOc blends were much larger than those in the corresponding ratio iPP/PEOc blends.

The complex viscoelastic behaviour of droplet-matrix structure blends has been quantitatively described by Palierne’s emulsion model [54]. The complex modulus of a blend can be predicted as a function of dispersed particle size, interfacial tension and complex modulus of each component in the blends. Under the assumptions that the deformation of inclusions remains small, within the limits of linear viscoelasticity, and that the dispersed droplets are nearly monodisperse, the linear complex shear modulus of the blend can be expressed as follows [59]:

(4) $$Gb^{*}=G_{m}^{*}\frac{1+3\phi H}{1-2\phi H}$$

**Figure 12:**
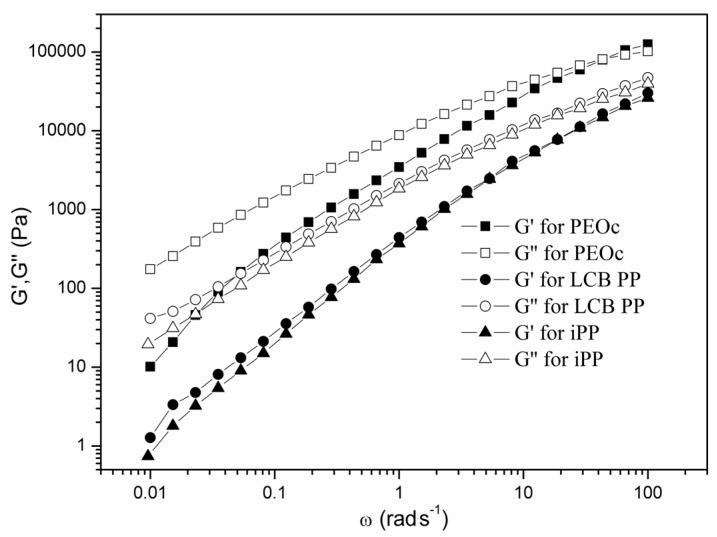
The frequency dependence of G’ and G” for the neat iPP, LCB PP and PEOc at 180 °C.

**Figure 13:**
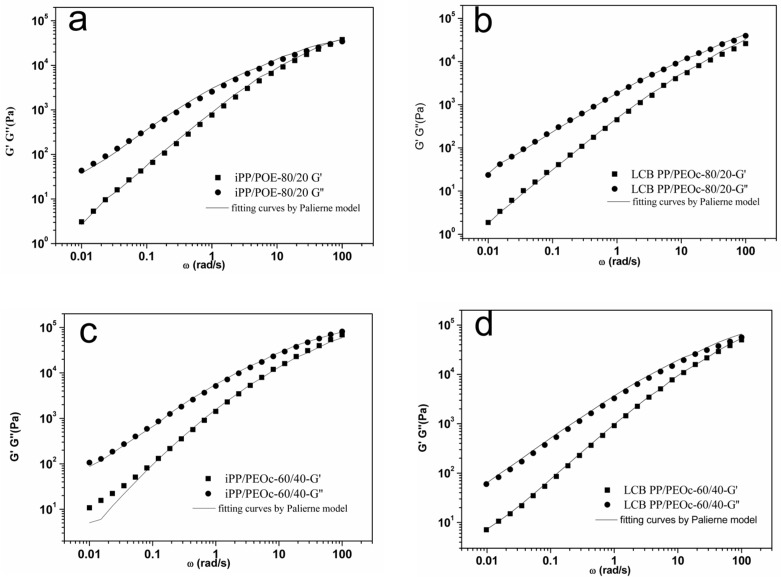
Palierne model fitting curves for the experimental dynamic moduli of the (**a**) iPP/PEOc-80/20 blend; (**b**) LCB PP/PEOc-80/20; (**c**) iPP/PEOc-60/40 blend; and (**d**) LCB PP/PEOc-60/40 blend at 180 °C.

(5) with H=(4τR)(2Gm*+5Gd*)+(Gd*−Gm*)(16Gm*+19Gd*)(40τR)(Gm*+Gd*)+(2Gd*+3Gm*)(16Gm*+19Gd*)

where $G_{b}^{*}$, $G_{m}^{*}$, and $G_{d}^{*}$ are the complex moduli of the blend, the matrix, and the dispersed phase, respectively. Additionally, *ϕ* denotes the volume fraction of the dispersed phase component, *τ* is the interfacial tension between the components of the blend, and *R* is the volume average radius of the dispersed phase. The interfacial tensions of the iPP/PEOc blend and the LCB PP/PEOc blend can be estimated from this model, using the complex modulus determined from dynamic rheological measurements and the size information of the dispersed phase estimated from SEM observations, respectively. The calculated *G’* and *G”* for neat iPP, LCB PP, and PEOc at 180 °C are shown in Figure 12. The Palierne model was found to fit well the experimental results, for the iPP/PEOc-80/20 and the LCB PP/PEOc blends as shown in Figure 13. The estimated values of the parameters are listed in Table 2. Generally the Palierne model assumes a dilute blend of non-interacting droplets. 40 wt % of the dispersed phase is a relatively large quantity which, for sure, will suffer from the interactions between droplets, coalescence and time dependency. Therefore, the fitting curve for iPP/PEOc-60/40 blend deviated from experimental data at low frequency. The interfacial tension for iPP/PEOc-80/20 at 180 °C was determined to be 0.67 mN m^−1^ (shown in Table 2). Carriere et al. reported that the interfacial tension for the PP/PEOc (with 24 wt % octene) blend was 0.56 ± 0.07 mN m^−1^ [60]. Kontopoulou et al. reported that the interfacial tension for the PP/PEOc (with 25.5 wt % octene) blend was 0.6 mN m^−1^ [61]. Our result is thus in good agreement with the values reported in literatures. 

Previous work by Tian et al. [7] indicated that LCB PP could be regarded as a blend of linear PP and star-shaped PP. The linear molecules in the blend possess comparable molecular mass with the linear molecules in the original PP. Moreover, the grafted star-shaped PP would have a longer relaxation time. Thus, they realized that the introduction of LCBs led to an increase in the *M*_w_ of the PP. The existence of LCBs may be assumed to lead to a slight decrease in compatibility between PP and PEOc, mainly due to the change in molecular architecture of PP from linear to star-shaped structures. The branching results in a significant increase in the *M*_w_ of the PP and complex structures on the PP backbones, which reduced the mobility of the LCB PP molecules. Consequently, this resulted in the increase of the interfacial tension between LCB PP and PEOc. In our case, an increase in the interfacial tension was found for LCB PP/PEOc (0.73–0.75 mN m^−1^, shown in Table 2) when compared with that of iPP/PEOc (0.67 mN m^−1^). For a two-component blend, when the *M*_w_ of one component is fixed, the effect of the *M*_w_ of another component on the interfacial tension, *τ,* can be described by the following empirical expression [6,62]:

*τ* = *τ_∞_* − *C* × *M_n_*^−z^ (6)

where *M*_n_ is the number average molecular weight, *τ_∞_* refers to the limiting value of the interfacial tension for the infinite *M*_n_ and *C* and *z* are constants with positive values. *C* reflects the dependence of interfacial tension on *M*_n_, and *z* varies from 0.5 to 1. According to Equation (6), the value of *τ* increases with *M*_n_. Accordingly, it could be assumed that the enlargement of PP chains resulted in an increased value of *τ* for the LCB PP/PEOc blends. 

**Scheme 1:**
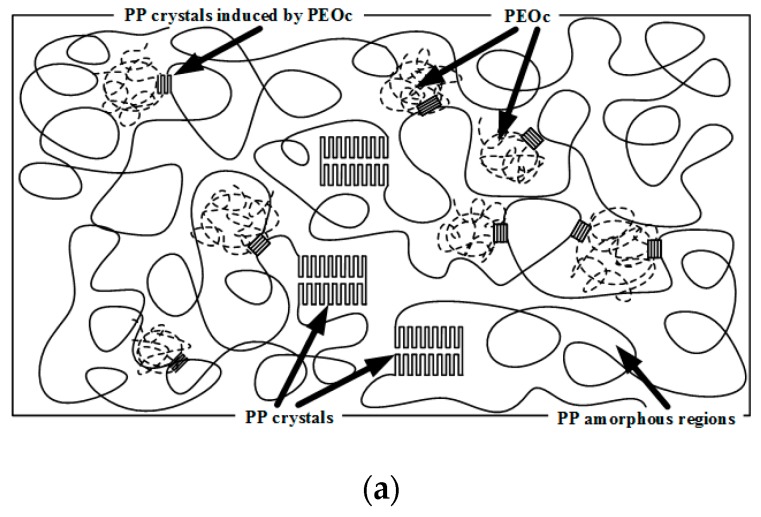

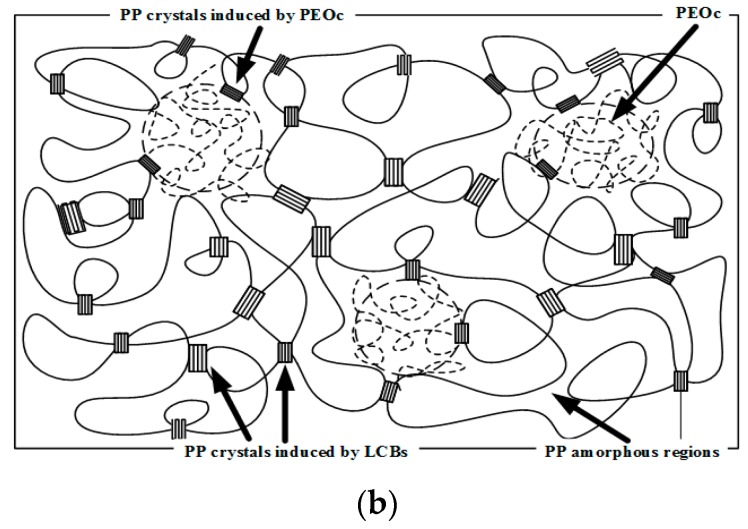
Schematic illustration of the crystallization patterns for (**a**) iPP/PEOc blends; and (**b**) LCB PP/PEOc blends.

Svoboda et al. [43] have reported that, in the case of the iPP/PEOc blends, PP spherulites would always find a bridge or way around obstacles (PEOc particles) to continue the growth of the spherulites, even if the spherulites were disturbed and became imperfect. The larger the obstacle, the longer was the time required for the spherulites to go around the obstacle, thus the slower the crystallization kinetics. In our previous study we reported that the presence of PEOc tends to promote the iPP chain to nucleate at the interface [45]. At the same weight ratio, the decrease in PEOc particle size would increase the interface area and improve the crystallization kinetics due to nucleation effects on the surface of PEOc. However, in the case of the LCB PP/PEOc blends, LCB PP crystallized faster than iPP in the iPP/PEOc blends, even if the size of PEOc particles was larger than that in the iPP/PEOc blends at the same weight ratio. This could be attributed to the existence of the large amount of LCBs in the blends, which acted as highly efficient nucleation agents, thus overcoming the shortage in the areas of the PP/PEOc interface. A possible crystallization mechanism for the LCB PP/PEOc and the iPP/PEOc blends is depicted in Scheme 1. In the iPP/PEOc blends, PEOc induces the nucleation of PP at the interface, and PP spherulites will grow out gradually into a relatively perfect structure in the matrix phase when the PEOc content is lower than 20%. In the case of the LCB PP/PEOc blends, the spherulite nuclei are induced predominantly by the LCBs, and the PP spherulites formed are imperfect due to the high crystal nuclei density. The nucleation effects of PEOc still exist, though it is not dominant during the whole crystallization process compared with that of the LCBs.

## 4. Conclusions

The crystallization behaviour of the LCB PP/PEOc blends with different weight ratios was investigated. A possible crystallization mechanism was proposed. During isothermal crystallization process, LCB PP/PEOc blends had a much higher crystal nuclei density and a shorter crystallization process than the iPP/PEOc blends. The crystallization kinetics of the LCB PP/PEOc blends was significantly enhanced by the LCBs. As efficient nucleation agents, the existence of LCBs dominated the crystallization kinetics of the LCB PP/PEOc blends. As for the non-isothermal crystallization process, the crystallization temperature of the LCB/PEOc blends was also increased with the PEOc content until it reached 60 wt %, indicating that the addition of PEOc accelerated the nucleation process due to heterogeneous nucleation at PEOc surfaces. However, higher content of PEOc (>60 wt %) inhibited the growth of the spherulites due to the steric hindrance. Flow-induced crystallization behaviour of the LCB PP/PEOc blends was also observed in steady shear rheological experiments. SEM observations showed that the LCB PP/PEOc blends exhibited a typical droplet-matrix structure when the PEOc content was less than 60 wt %. Based on the SEM and dynamic complex modulus information, the interfacial tension for the LCB PP/PEOc at 180 °C was estimated to be 0.73–0.75 mN m^−1^, larger than that of 0.67 mN m^−1^ for the iPP/PEOc, reflecting the influence of the *M*_w_ of LCB PP on the interfacial behaviour in the blend.

## Figures and Tables

**Table 1:** *t*_1/2_ and Avrami parameters for the iPP/PEOc and LCB PP/PEOc blends undergoing isothermal crystallization at 140 °C.

Sample	*t*_1/2_ (s)	*n*	*k* × 10^9^ (s^−1^)
iPP/PEOc-80/20	2556	2.35	7.85
iPP/PEOc-40/60	2712	2.33	5.67
LCB PP/PEOc-80/20	83	2.80	3371
LCB PP/PEOc-40/60	87	2.77	2529

**Table 2:** Estimated values of the parameters of the Palierne model for iPP/PEOc and LCB PP/PEOc blends at 180 °C.

Sample	*ϕ*	*R* (μm)	*τ* (mN m^−1^)
iPP/PEOc-80/20	0.208	0.7	0.67
iPP/PEOc-60/40	0.411	0.98	0.65
LCB PP/PEOc-80/20	0.208	1.2	0.75
LCB PP/PEOc-60/40	0.411	3.5	0.73
